# Folliculin Controls the Intracellular Survival and Trans-Epithelial Passage of *Neisseria gonorrhoeae*

**DOI:** 10.3389/fcimb.2020.00422

**Published:** 2020-09-04

**Authors:** Tao Yang, Motaharehsadat Heydarian, Vera Kozjak-Pavlovic, Manuela Urban, Richard P. Harbottle, Thomas Rudel

**Affiliations:** ^1^Biocenter, Chair of Microbiology, University of Würzburg, Würzburg, Germany; ^2^DNA Vector Lab, German Cancer Research Centre (DKFZ), Heidelberg, Germany

**Keywords:** gonococcal invasion, folliculin, autophagy, polarized epithelium, polarized cell culture

## Abstract

*Neisseria gonorrhoeae*, a Gram-negative obligate human pathogenic bacterium, infects human epithelial cells and causes sexually transmitted diseases. Emerging multi-antibiotic resistant gonococci and increasing numbers of infections complicate the treatment of infected patients. Here, we used an shRNA library screen and next-generation sequencing to identify factors involved in epithelial cell infection. Folliculin (FLCN), a 64 kDa protein with a tumor repressor function was identified as a novel host factor important for *N. gonorrhoeae* survival after uptake. We further determined that FLCN did not affect *N. gonorrhoeae* adherence and invasion but was essential for its survival in the cells by modulating autophagy. In addition, FLCN was also required to maintain cell to cell contacts in the epithelial layer. In an infection model with polarized cells, FLCN inhibited the polarized localization of E-cadherin and the transcytosis of gonococci across polarized epithelial cells. In conclusion, we demonstrate here the connection between FLCN and bacterial infection and in particular the role of FLCN in the intracellular survival and transcytosis of gonococci across polarized epithelial cell layers.

## Introduction

*Neisseria gonorrhoeae* is a Gram-negative diplococcus that causes the sexually transmitted disease (STD) gonorrhea. Gonorrhea is the second most frequently reported STD, which can lead to pelvic inflammatory disease and infertility. Asymptomatic infection is common and if untreated, the infection may spread to the rest of the body triggering disseminated gonorrhea. Currently, *N. gonorrhoeae* developed resistance to virtually all the available antibiotics used for treatment. Vaccine development has been hampered by the enormous variety of gonococcal surface factors. Identification of the host factors involved in the host-pathogen interaction is a crucial step in understanding the disease development and uncovering novel therapeutic approaches.

*N. gonorrhoeae* has evolved intricate mechanisms to evade the host immune attack. The antigenic and phase-variable pili confer the initial attachment to the epithelial cell (McGee et al., 1981; Rudel et al., 1992), but the switch to a non-piliated status has been suggested as a prerequisite for efficient invasion (Faulstich et al., 2013). The phase-variable opacity-associated (Opa) adhesion proteins are other major surface structures of *N. gonorrhoeae*, mediating adherence to and entry into host cells (Makino et al., 1991; Kupsch et al., 1993; Chen and Gotschlich, 1996; Bos et al., 1997a,b; Gray-Owen et al., 1997). Opa proteins recognize different classes of cellular receptors including members of the carcinoembryonic antigen cell adhesion molecule (CEACAM) family (Virji et al., 1996; Gray-Owen, 1997; Gray-Owen et al., 1997) and heparin sulfate proteoglycans (HSPGs) (Chen, 1995; van Putten and Paul, 1995) which enables *N. gonorrhoeae* to invade epithelial cells and interact with neutrophils and lymphocytes. A large body of *in vitro* studies revealed that the efficiency of HSPG-mediated invasion was enhanced by vitronectin, fibronectin or fetal bovine serum (FBS) (Dehio et al., 1998; Duensing and van Putten, 1998; van Putten, 1998). In addition to pili and Opa, the serotype A of the major outer membrane porin (PorB_IA_) induces *N. gonorrhoeae* infection and invasion under low phosphate condition (Kuhlewein et al., 2006). PorB_IA_-expressing gonococci are frequently isolated from patients with severe disseminating infection (Morello and Bohnhoff, 1989).

A recent study indicated that intracellular *N. gonorrhoeae* were targeted to the autolysosome and destroyed. A small portion of the bacteria escaped the host eradication by downregulating CD46-cyt1-mediated autophagy flux (Kim et al., 2019). Besides, *N. gonorrhoeae* secretes IgA protease, which cleaves lysosomal-associated membrane protein 1 (LAMP1) responsible for lysosome integrity, to increase their survival chance (Lin et al., 1997). Furthermore, *N. gonorrhoeae* stimulates apoptosis of the host cell in a Bak/Bax-dependent manner to accomplish infection as well (Muller, 2002; Kepp et al., 2007).

While two-dimensional flat monolayers have provided meaningful insight into host-pathogen interactions, they lack many essential features of the native infection microenvironment. Naturally, *N. gonorrhoeae* infects the mucosal surfaces of the female cervix and the male urethra, anorectal, pharyngeal and conjunctival mucosa. These mucosal surfaces are made of either multilayered non-polarized squamous epithelial cells or monolayered squamous epithelial cells (Wira et al., 2005). The epithelium is sealed by the adherence junction, which contains catenins and E-cadherin (E-cad) and the tight junction, containing claudin, occluding and zonula occludens (ZO) proteins 1 and 2 and others, to prevent the transmigration of pathogens and toxins (Miyoshi and Takai, 2005; Hartsock and Nelson, 2008; Niessen and Gottardi, 2008). Studies using polarized cell culture models developed on Transwell® inserts, rotating wall vessel bioreactor and porcine small intestinal submucosa (SIS) scaffold, respectively showed the disruption of apical junctions upon *N. gonorrhoeae* infection (Stein et al., 2015; Łaniewski et al., 2017; Heydarian et al., 2019).

The present study was designed to determine the role of Folliculin (FLCN) in *N. gonorrhoeae* infection. FLCN, a 64 kDa protein, is associated with Birt-Hogg-Dubé syndrome, an autosomal dominant condition featured by dermatologic lesions, pulmonary manifestations, and renal tumors (Skolnik et al., 2016). FLCN was proposed to function as tumor suppressor active in several signaling pathways (Schmidt and Linehan, 2018). In addition, FLCN has been implicated in the activation of Rab GTPases as guanine nucleotide exchange factor, such as Rab11A and Rab34, which are involved in vesicular trafficking (Starling et al., 2016; Zhao et al., 2018) and has been suggested to control cellular E-cadherin protein levels and polarization in a positive (Nahorski et al., 2012) and negative (Medvetz et al., 2012) manner. Furthermore, FLCN was also reported to be involved in autophagy regulation as a negative regulator of AMP-activated protein kinase (AMPK) interfering with the autophagy flux (Possik et al., 2014) and as an interaction partner of the crucial autophagy initiator protein LC3/GABARAP (Dunlop et al., 2014).

In this study, we demonstrate that FLCN is not important for the adherence or invasion of *N. gonorrhoeae*, but plays the role in the intracellular survival of gonococci probably by modulating the autophagic flux. Furthermore, FLCN interferes with the amount and polarization of E-cadherin. Knockdown of E-cadherin reduces the cellular autophagy and increases the *N. gonorrhoeae* survival.

## Materials and Methods

### *N. gonorrhoeae* Strains

The *N. gonorrhoeae* MS11 strain derivatives N924 (PorB_IB_, Opa^−^, Pili^−^) and N931 (PorB_IB_, Opa_50_, Pili^−^) were grown on GC agar plates (Gibco/Thermo Fisher Scientific, Massachusetts, USA) supplemented with 1% vitamin mix for 14–17 h at 37 °C in 5% CO_2_. Opa phenotypes were selected by colony morphology and verified by Western blot.

### Cell Culture, Gene Knockdown, Gene Overexpression

HeLa2000 cells were cultured in Roswell Park Memorial Institute medium (RPMI) (Gibco/Thermofisher scientific, Massachusetts, USA), Hec-1-B (human endometrial adenocarcinoma cell line; ATCC^®^ HTB113™) were cultured in Dulbecco's Modified Eagle Medium (DMEM) (Gibco/Thermofisher scientific, Massachusetts, USA). UOK257 is a cell line derived from the renal tumor of a Birt-Hogg-Dubé syndrome (BHD) patient and deficient in FLCN expression (Yang et al., 2008). In the UOK-FLCN cell line, FLCN was stably expressed by the introduction of a pCAG-FLCN-SMAR plasmid, a refined version of the FLCN-SMAR plasmid originally described (Wong and Harbottle, 2013) (as depicted in Plasmid map Figure S1). After transfection of UOK257 cells with this vector using XtremeGene9, the transgenic cells were selected using 0.5μg/ml puromycin for a period of 2 weeks. FLCN transgene expression was then stably maintained in these cells without the use of antibiotics. UOK257 and UOK-FLCN cells were cultured in DMEM medium. All media were supplemented with 10% FBS (Sigma/Merck, Darmstadt, Germany) and 1% Penicillin/Streptomycin (Gibco/Thermofisher scientific, Massachusetts, USA). The following shRNAs were used to produce knockdown cell lines: shFLCN (5′-TCAGTATGCAGTCGCAATAAC), shEcad_1 (5′-GGACGTGGAAGATGTGAAT-3′) and shEcad_2 (5′-GTCTAACAGGGACAAAGAA-3′). Lentiviral shRNA constructs targeting the FLCN or E-cadherin gene were generated by inserting shRNA oligonucleotides into the ClaI and MluI sites of the PLVTHM vector. GFP-expressing cells were sorted by FACS. The quantitative polymerase chain reaction (qPCR) and Western blot were used to select the positive knockdown cells. The qPCR primers used for FLCN were 5′-GCCAGTCTTCAAGTCCCTCC-3′ and 5′-TGTATGGGATGATGCGGACG-3′. The primers for E-cadherin were 5′-CCCGCCTTATGATTCTCTGCTCGTG-3′ and 5′-TCCGTACATGTCAGCCAGCTTCTTG-3′.

### Infection Experiments

Cells were grown in 24-well cell culture plates with or without glass coverslips to 60–70% confluency. Infections were performed in DMEM or RPMI medium supplemented with 10% FBS and the process was modified from the previous description (Kuhlewein et al., 2006). The 60–70% confluence monolayers were transferred to fresh infection medium. Bacteria were suspended in HEPES medium and added to the cells at a multiplicity of infection (MOI) of 50. The infected samples were centrifuged for 5 min at 120 × g to synchronize infection and incubated at 37°C in 5% CO_2_ humidified atmosphere for 2 h. The infections were stopped by washing the cells three times with infection medium. Bafilomycin A1 (BafA1, Sigma-Aldrich/Merck, St. Louis, USA) treated samples were incubated with 5 nM BafA1 for 16 h before infection.

### Quantification of Adherence, Invasion and Survival *N. gonorrhoeae*

To quantify total cell-associated bacteria, infected cells were lysed in infection medium containing 1% saponin (Sigma-Aldrich/Merck, St. Louis, USA) for 7 min at 37°C and 5% CO_2_. Serial dilutions were plated on GC-agar plates and colony-forming units (CFU) were counted after 24 h. To quantify the survival of intracellular bacteria, infected cells were incubated in infection medium in the presence of 50 μg/ml gentamicin for another 2 h to kill the extracellular bacteria. Bacteria were released from the infected cell by saponin lysis and CFUs were determined. Bacterial adherence was obtained by subtracting the number of intracellular surviving bacteria from the total cell-associated bacteria. Another method for determining intracellular bacteria was the differential immunostaining as described by Kuhlewein et al. (2006). Overlay images of single channels were obtained using FIJI. Double-positive staining specified extracellular and single-positive staining intracellular bacteria.

### Immunoblotting Analyses

Cell lysates were analyzed by sodium dodecyl sulfate (SDS)-poly-acrylamide gel electrophoresis (PAGE). Proteins were transferred to polyvinylidene difluoride membranes (Millipore, Massachusetts, USA) and then incubated with different antibodies. The primary antibodies used in this study were FLCN (Cell signaling, Massachusetts, USA), E-cadherin (Proteintech, Manchester, United Kingdom), LC3B (Cell signaling, Massachusetts, USA).

### Generation of Transwell Infection Models

Transwell models were developed based on UOK-FLCN and UOK257 cell lines for bacterial infection using the following procedure: 6.5 mm diameter, 3 μm pore size polyester Transwell inserts (Corning, Massachusetts, USA) were seeded with 100,000 epithelial cells on the apical side. Models were grown 10 days under submerged static conditions at 37°C/5% CO_2_ for further experiments. The integrity of the monolayer was determined using 4 kDa FITC-dextran (Sigma-Aldrich/Merck, St. Louis, USA) permeability assay as described before (Heydarian et al., 2019).

### *N. gonorrhoeae* Infection of Transwell Infection Models

Infections of cells grown on Transwell inserts were performed in DMEM plus 10% FBS at MOI50. To determine the transmigration of bacteria across the polarized monolayer after different time points of infection, 25 μl of medium were plated on GC-agar plate or the medium was centrifuged shortly at 2,400 × g and the pellet resuspended in 25 μl of medium was plated on GC agar as described (Heydarian et al., 2019). Infected or non-infected samples were fixed with 4% PFA at RT for 30 min and decorated with anti-E-cadherin and anti-ZO-1 (Proteintech, Manchester, United Kingdom) to observe cell junction proteins and polarization. Z-stack images were taken using an SP5 confocal microscope and reconstructed by FIJI.

### Statistical Analysis

Statistics were performed using Student's *t*-test in the R package or using One-Way ANOVA, Tukey's multiple comparison test in GraphPad Prism Software (GraphPad Software, Inc., San Diego, USA). Data are represented as means ± s.d, *P* < 0.05 was taken as statistical significance.

## Results

### FLCN Is a Host Factor Critical for Intracellular *N. gonorrhoeae*

**Figure 1:**
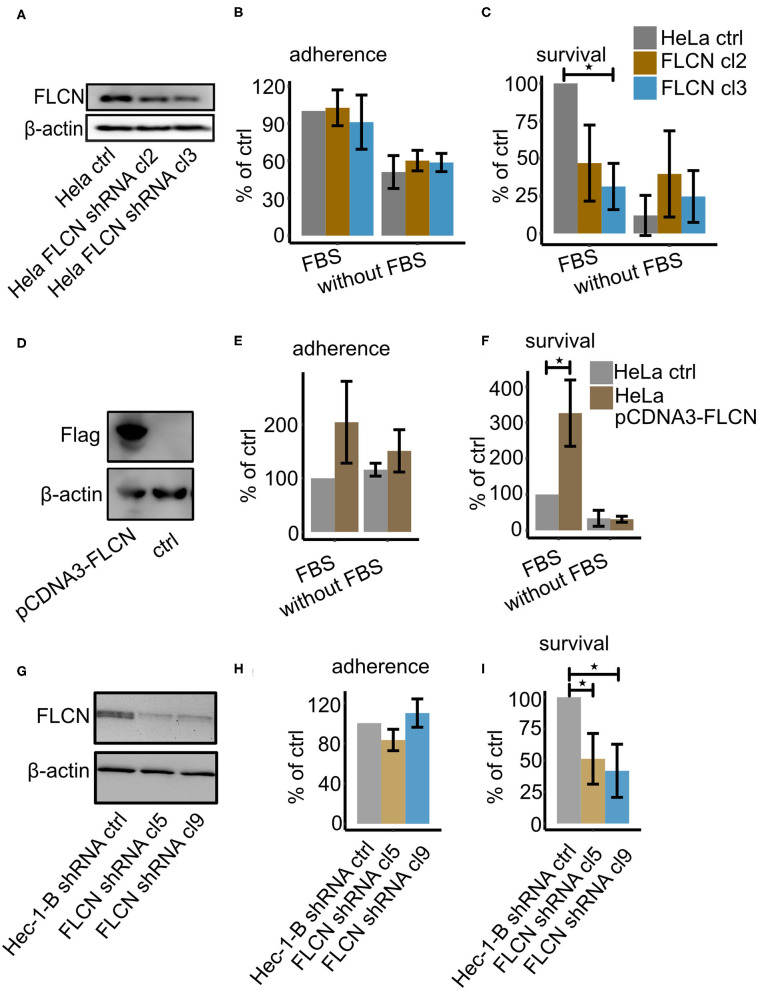
FLCN downregulation leads to decreased survival of gonococci. HeLa2000 and Hec-1-B cells and their respective knockdown cell clones were infected with N931 (MOI 50) in RPMI or DMEM medium with or without FBS and then subjected to a gentamicin protection assay. **(A)** Western blot of FLCN of HeLa control and FLCN knockdown cells. **(B)** Infected cells were lysed and the total number of colonies forming units was quantified by dilution plating. Shown is the number of adhered bacteria (calculated as total minus surviving bacteria). **(C)** 50 μM gentamicin was added to the infected cells for 2 h to kill the extracellular gonococci. Cells were lysed and the number of colonies forming units was quantified. **(D)** Western blot of control and FLCN-Flag overexpressing HeLa2000 cells. Adhered **(E)** and intracellular **(F)** gonococci were determined and calculated by normalizing to HeLa control plus FBS. **(G)** Western blot of control and shRNA-expressing Hec-1-B cells. Adherent **(H)** and intracellular **(I)** bacteria were determined and normalized to control shRNA expressing Hec-1-B in DMEM plus FBS. Data **(B,C,E,F,H,I)** represent the mean ± s.d with three independent repeats. Significance was determined using student *t*-test, ^*^P < 0.05.

By orchestrating autophagy signaling (Dunlop et al., 2014; Possik et al., 2014) and cell junction integrity (Medvetz et al., 2012; Nahorski et al., 2012; Goncharova et al., 2014), FLCN plays a central role in two central processes connected to mucosal infection with *N. gonorrhoeae*. To analyze the function of FLCN in gonococcal infection, we generated a stable shRNA-expressing HeLa2000 cell line where FLCN expression was downregulated (Figure 1A). The knockdown efficiency was about 50% and 40% (qPCR) for two selected clones. These cell lines were then used for gentamicin protection assays to determine the number of adherent and intracellular bacteria after infection. Cells were infected with N931 (Opa50, PorB_1B_, Pili^−^) in both RPMI and RPMI with FBS conditions. In general, the number of adherent and gentamicin-resistant gonococci was higher in the presence of FBS in the infection medium, indicating that the presence of FBS enhanced bacterial adherence and uptake. FLCN downregulation did not affect adherence (Figure 1B), but dramatically reduced the number of intracellular gonococci (Figure 1C) in the presence of FBS. The effect of FBS affects the interaction of Opa50 expressing bacteria with host cell integrin receptors (Dehio et al., 1998; Duensing and van Putten, 1998; van Putten, 1998), whereas FLCN expression has been shown to be independent of FBS (Goncharova et al., 2014) and growth factors (Laviolette et al., 2017). To confirm that the presence of intracellular bacteria depended on FLCN, we overexpressed FLCN in HeLa2000 cells (Figure 1D). As expected, the overexpression of FLCN did not affect the adherence significantly (Figure 1E) but increased the number of gentamicin resistant gonococcal (Figure 1F). Furthermore, similar results were obtained in Hec-1-B endometrial adenocarcinoma cells upon knockdown of FLCN (Figures 1G–I), excluding a cell-type-specific effect (Figure 1G). In summary, our data demonstrated that FLCN is an important factor that influences the number of intracellular gonococci in epithelial cells.

### FLCN Supports *N. gonorrhoeae* Intracellular Survival

**Figure 2:**
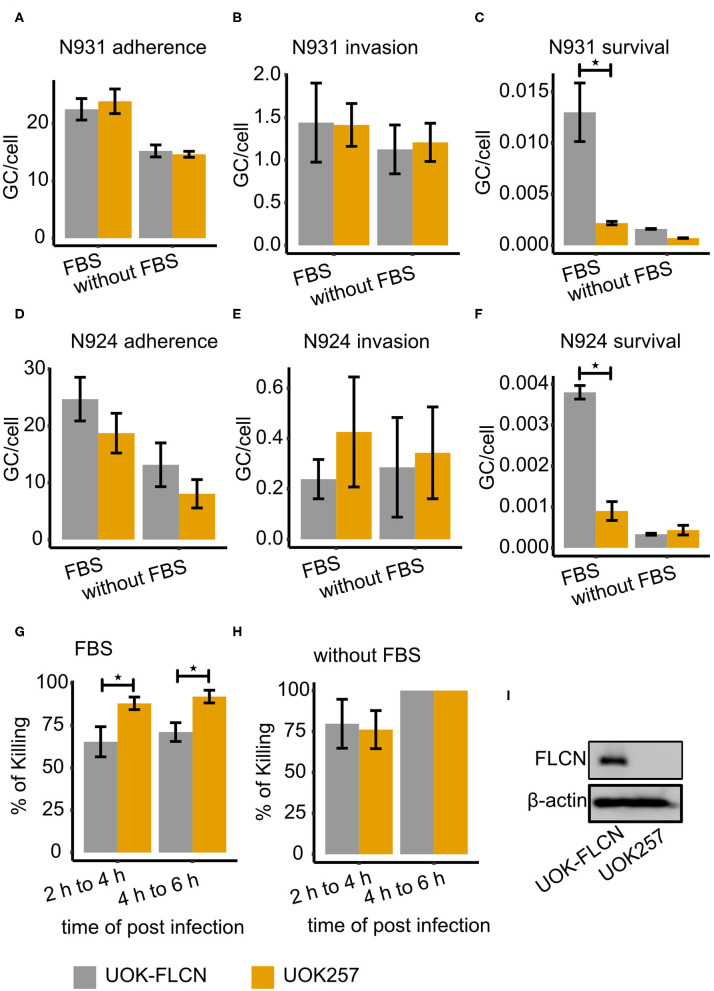
FLCN supports gonococcal intracellular survival. Gonococcal adherence and survival were determined by gentamicin protection assay and invasion by differential immunostaining (see methods for details). Strains N931 (pili^−^, ${\text{porB}}_{1\text{A}}^{-}$, Opa50^+^) and N924 (pili^−^, ${\text{porB}}_{1\text{A}}^{-}$, Opa50^−^) were used for infection. **(A–C)** Adherence, invasion and survival of N931 was determined in UOK-FLCN and UOK257 cells. **(D–F)** Adherence, invasion and survived of N924 in UOK-FLCN and UOK257 cells. **(G,H)** Shown are the rates by which intracellular N931 were killed in the respective cells grown in DMEM with **(G)** or without **(H)** FBS. Data represent the mean ± s.d with three independent repeats. Significance was determined using student *t*-test, ^*^P < 0.05. **(I)** Western blot of UOK-FLCN and UOK257 to detect FLCN and actin was a loading control.

To investigate whether the increased numbers of gentamicin-resistant gonococci in the presence of FLCN was a consequence of better invasion or impaired intracellular killing, we investigated adherence, invasion and survival by differential immunofluorescence staining and gentamicin protection assay in parallel (Rechner et al., 2007). One set of the infected samples was treated with saponin to quantify the total cell-associated bacteria. The second set was incubated with gentamicin for another 2 h to kill the extracellular bacteria and intracellular survival of bacteria was determined in CFU assays. Adherence was calculated as the total number of bacteria minus the number of intracellular survivors. The third set of samples was fixed and stained twice with different anti-gonococcal antibodies. The first staining was done before permeabilizing the host cell membrane, the second was carried out after the permeabilization. Hence, invaded bacteria were only labeled once with antibody whereas extracellular bacteria were double labeled. Confocal images were taken, and invasion was quantified by FIJI analysis. These assays were performed with UOK257, a FLCN-deficient cell line and the FLCN-complemented cell line UOK-FLCN. FLCN did not affect adherence or invasion but contributed to the survival of N931 (Figures 2A–C). In addition, to determine whether this effect depended on the surface adhesin proteins, we used the Opa- and pilus-negative derivative N924 (Opa^−^, PorB_1B_, Pili^−^) as a control. FLCN did not affect adherence (Figure 2D) or invasion (Figure 2E), but was critical for the survival of N924 (Figure 2F), although the efficiency of gonococcal uptake was clearly reduced compared to the bacteria expressing Opa-adhesins. Taken together the data of N931 and N924, we assumed that FLCN played a general role in gonococcal survival. Furthermore, we could show that the efficiency by which cells could kill engulfed N931was lower in UOK-FLCN than in UOK257 with FBS in the infection medium (Figure 2G) whereas the killing efficiency was similar in UOK-FLCN and UOK257 in DMEM condition (Figures 2H,I). Thus, FLCN favors gonococcal survival by decreasing the host killing efficiency. This effect is visible, however, only in the presence of FBS during the infection.

### FLCN-Mediated Downregulation of Autophagy Supports the Survival of Intracellular Gonococci

**Figure 3:**
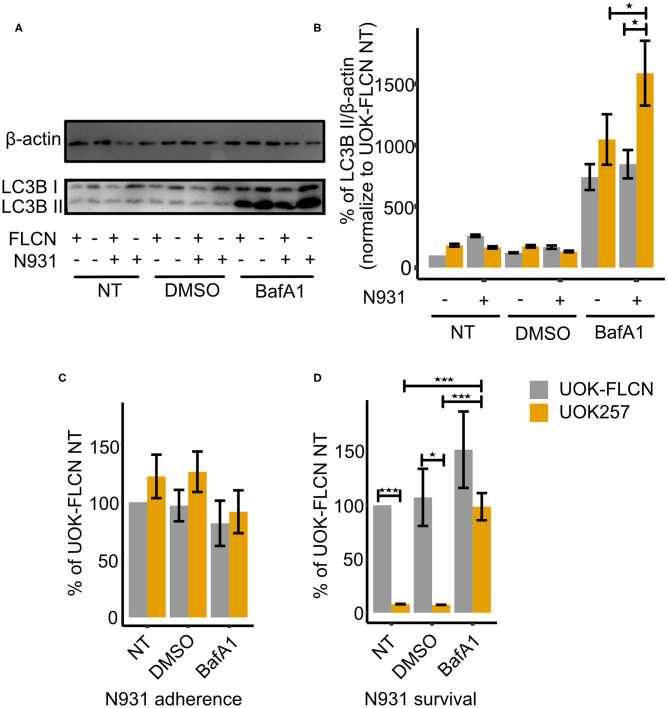
FLCN inhibits the autophagy flux and supports gonococcal intracellular survival. **(A)** Representative immunoblot displaying LC3B-I, LC3B-II, and β-actin of UOK-FLCN and UOK257 cells. The cells were either left untreated or were treated with the solvent DMSO or with 5 nM Bafilomycin A1 (BafA1) for 16 h in the absence or presence of gonococcal infection. **(B)** Densitometric quantification of LC3-II levels in three independent immunoblots shown in **(A)**. Data represent the mean ± s.d with three independent repeats. Significance was determined using One-Way ANOVA, ^*^P < 0.05. **(C)** Adherence and **(D)** intracellular survival of N931 were determined in UOK-FLCN and UOK257 either left untreated or treated with DMSO or 5 nM BafA1 for 16 h. The cells were infected with N931 (MOI 50) for 2 h and then analyzed by gentamicin protection assay. Data represent the mean ± s.d with three independent repeats. Significance was determined using student *t*-test, ^*^P < 0.05, ^***^P < 0.001.

As reported previously (Kim et al., 2019), *N. gonorrhoeae* modulates the autophagy flux to evade intracellular killing. In addition, FLCN has been implicated in the regulation of autophagy as a direct interaction partner for LC3/GABARAP (Dunlop et al., 2014). During autophagy the cytosolic LC3-I conjugates to phosphatidylethanolamine to form LC3-II, which is recruited to autophagosomal membranes. LC3-II is degraded by lysosomal enzymes upon the fusion of the lysosome with the autophagosome (Yoshii and Mizushima, 2017). Blocking LC3-II degradation by Bafilomycin A1 (BafA1) reflects the total amount of LC3-II. As shown in Figures 3A,B, FLCN inhibits the autophagy as the total amount of LC3B II of UOK257 tends to be higher than that of the UOK-FLCN. Additionally, FLCN inhibits autophagy upon gonococcal infection. The total amount of LC3B-II has increased significantly in the FLCN negative cell (UOK257) while that in the FLCN positive cell (UOK-FLCN) was not significantly increased. Therefore, we assume that FLCN inhibits the autophagic flux, which supports the survival of intracellular gonococci.

To test our hypothesis, we carried out gentamicin protection assays in non-treated cells, solvent (DMSO)- and BafA1-treated cells. As shown in Figure 3C, gonococcal adherence was similar for UOK-FLCN and UOK257 under all treatment conditions. In contrast, bacterial survival was strongly increased in BafA1-treated UOK cells (Figure 3D). These data supported our hypothesis that FLCN was critical for gonococcal survival by interfering with the autophagic flux.

### FLCN Negatively Regulates E-cadherin Polarization

**Figure 4:**
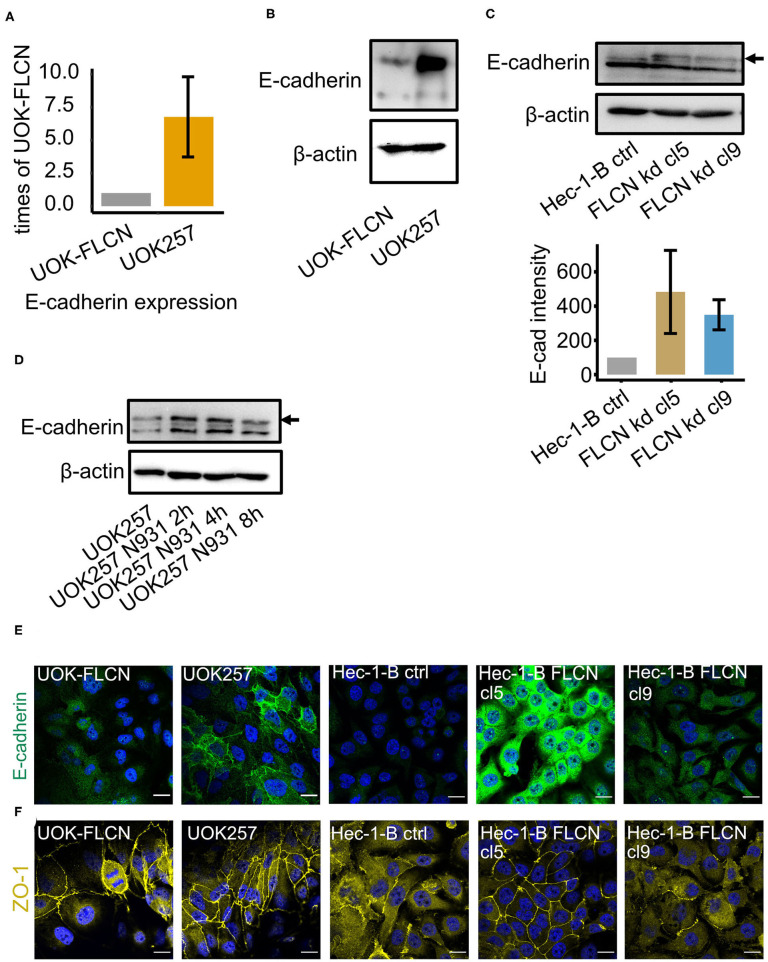
FLCN negatively regulates the E-cadherin in 2D cell culture. **(A)** qPCR of E-cadherin in UOK-FLCN and UOK257 cells. GAPDH was used as an internal control. **(B)** Western blot of UOK-FLCN and UOK257 cells to detect E-cadherin. Actin served as a loading control. **(C)** Western blot of the control and FLCN-knockdown Hec-1-B cells to detect E-cadherin and actin as a loading control. **(D)** UOK257 were infected with N931 for different time points and E-cadherin was analyzed by Western blotting. **(E)** Immunofluorescence staining of E-cadherin in UOK-FLCN, UOK257 and the control and FLCN knockdown cells of Hec-1-B. **(F)** Immunofluorescence staining of ZO-1 in UOK-FLCN, UOK257 and the control and FLCN knockdown cells of Hec-1-B.

Autophagy influences cell polarization probably by the control of cell junction protein expression (Nighot and Ma, 2016). Similarly, FLCN has been shown to control cellular E-cadherin protein levels and polarization in a positive (Nahorski et al., 2012) and negative (Medvetz et al., 2012) manner. We, therefore, investigated the role of FLCN in cellular polarization and in particular the fate of the adherence junction protein E-cadherin in gonococcal infection. In UOK-FLCN cells, we found lower levels of E-cadherin mRNA and protein compared to UOK257 (Figures 4A,B). To rule out the possibility that this was a cell-type-specific phenomenon, we tested single clones of Hec-1-B FLCN knockdown cells (Figure 1G) for E-cadherin expression (Figure 4C). E-cadherin expression was very low in control knockdown cells but detectable in Hec-1-B FLCN knockdown cells (Figure 4C), supporting the role of FLCN in suppressing E-cadherin expression. During gonococcal infection, E-cadherin protein levels were transiently increased (Figure 4D). Different from the adherence junction protein E-cadherin, the tight junction protein ZO-1 was not influenced by the presence or absence of FLCN in UOK cell (Figures 4E,F, **6A**, **7A**), indicating that FLCN influenced selectively adherence junctions.

### E-cadherin Knock-Down in UOK257 Increases *N. gonorrhoeae* Survival

**Figure 5:**
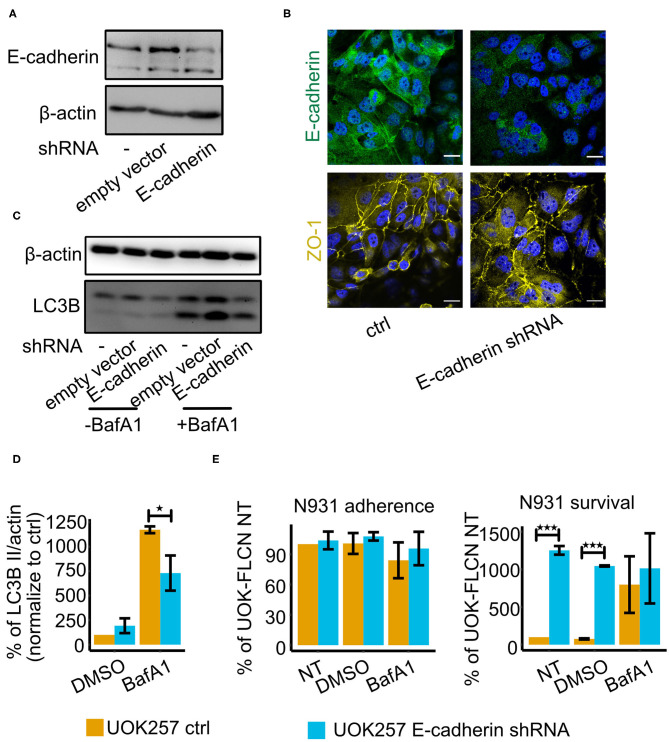
E-cadherin knockdown in UOK257 cell increases gonococcal survival in 2D cell culture. **(A)** Western blot of E-cadherin in non-treated, empty vector-transfected and E-cadherin shRNA-transfected UOK257 cells. **(B)** Immunofluorescence staining of E-cadherin and ZO-1 in the control and E-cadherin knockdown cells of UOK257. **(C)** Representative immunoblot displaying LC3B-I, LC3B-II, and β-actin in the control and E-cadherin knockdown of UOK257 cells. The cells were treated with the solvent DMSO and 5 nM BafA1in DMSO for 16 h. **(D)** Densitometry quantification of LC3-II levels in three independent experiments [described in **(C)**]. LC3B-II in each lane was normalized to β-actin and the different experiment were normalized to the values obtained for the non-treated UOK257. Data represent the mean ± s.d with three independent repeats. Significance was determined using student *t*-test, ^*^P < 0.05. **(E)** UOK257 were transfected with control or E-cadherin shRNAs and either treated with DMSO or 5 nM BafA1 in DMSO for 16 h. Then these cells were infected with gonococci for 2 h and adherence (left) and survival (right) was determined. Data represent the mean ± s.d with three independent repeats. Significance was determined using student *t*-test, ^***^P < 0.001.

To elucidate the role of E-cadherin in gonococcal infection, we constructed an E-cadherin knockdown cell line in UOK257. The successful knockdown was shown by both Western blot and immunofluorescence staining (Figures 5A,B). To test whether E-cadherin had a role in autophagy flux regulation, we determined the LC3B II levels in both non-treated and BafA1-treated cells. These experiments revealed that autophagy was increased in the presence of E-cadherin (Figures 5C,D). In addition, a gentamicin protection assay was used to determine the adherence and survival of gonococci in control and E-cadherin knockdown cells. E-cadherin knockdown did not affect the adherence but increased the survival rate of gonococci (Figure 5E). To further investigate if the survival defect of E-cadherin was mediated by autophagy, we compared the survival of gonococci in mock-treated and BafA1-treated cells. The inhibition of autophagy counteracted the survival defects in the E-cadherin control cells but had no major effect on gonococcal survival in E-cadherin knockdown cells (Figure 5E), supporting a role of E-cadherin in autophagy regulation and suppression of gonococcal intracellular survival.

### FLCN Negatively Regulates E-cadherin and Delays Gonococcal Transmigration in Polarized Cells

**Figure 6:**
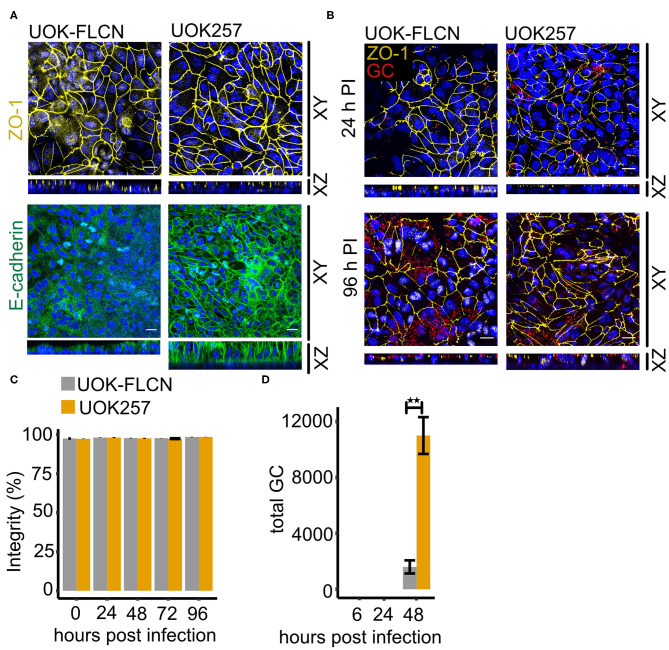
FLCN negatively regulates E-cadherin and it delays gonococcal transmigration in polarized epithelial cells. **(A)** Confocal microscopy of transwell models of UOK-FLCN and UOK257. The cells were cultured for 10 days and then fixed and stained for E-cadherin or ZO-1 antibodies and DAPI. Z-stacks were made and reconstructed using FIJI. Shown were Z-projections (XY) and orthogonal sections (XZ). **(B)** Confocal microscopy of N931 infected (MOI 50) transwell models at different time points. Shown were Z-projections (XY) and orthogonal sections (XZ). **(C)** Barrier integrity of N931-infected (MOI 50) UOK-FLCN and UOK257 models were measured by FITC-dextran assay (4 kDa). Data represent the mean ± s.d with three independent repeats. **(D)** Transmigration of N931 (MOI 50) determined in UOK-FLCN and UOK257 transwell models. Data represent the mean ± s.d with three independent repeats, ^**^P < 0.01.

To reach deeper tissues, gonococci have to overcome the epithelial barrier during infection, of which the cell-cell communication and cell polarization are critical markers (Wang et al., 2017; Heydarian et al., 2019). To investigate the role of FLCN in this process, we seeded the UOK cells on transwell support to induce cell polarization. Similar to the results in 2D cell culture, FLCN expression did not affect ZO-1 localization to the membrane, but it decreased the amount of membrane-associated E-cadherin and no polarization of E-cadherin was found in UOK-FLCN (Figure 6A).

To determine whether the defect of E-cadherin expression in UOK-FLCN cells affects gonococcal transmigration, we infected cells grown on transwells with N931 from the apical compartment and monitored transmigration by immunofluorescence microscopy and recovery of viable bacteria from the basal compartment of the transwell. Gonococci remained at the top of the cells even after 24 h infection time (Figure 6B). Bacteria transmigrated to the basal side of the epithelial layer 96 h post-infection (pi) in UOK257 but not in UOK-FLCN (Figure 6B). Despite unstructured ZO-1 staining after 96 h of infection, the barrier integrity investigated by FITC-dextran permeability assay remained similar as in the non-infected sample (Figure 6C). The delayed transmigration of gonococci in UOK-FLCN was also supported by a 7-fold higher CFU count of the bacteria collected from the bottom of the transwell in FLCN-negative cells at 48 h pi (Figure 6D), suggesting that gonococcal transmigration is inhibited in FLCN-positive cells.

### E-cadherin Inhibits Gonococcal Transmigration in Polarized Cells

**Figure 7:**
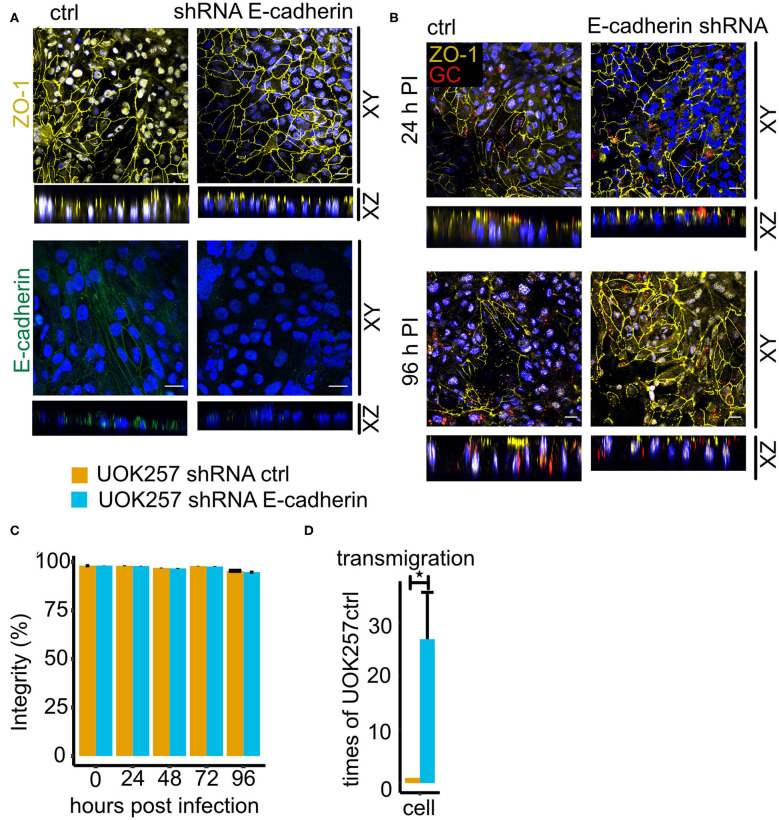
Loss of E-cadherin enhances gonococcal transmigration in polarized epithelial cells. **(A)** Confocal microscopy of control and E-cadherin knockdown UOK257 cells grown transwells. The samples were stained for ZO-1 or E-cadherin and DAPI. Z-stacks were generated and reconstructed with FIJI. Shown are XY projections and orthogonal sections (XZ). **(B)** Confocal microscopy of N931-infected (MOI 50) transwell models at different time points. Shown are Z-projections (XY) and orthogonal sections (XZ). **(C)** Barrier integrity of N931-infected (MOI 50) UOK257 cells transfected with the vector control pLVTHM and E-cadherin shRNA in pLVTHM. Barrier integrity was determined by FITC-dextran assay (4 kDa). Data represent the mean ± s.d with three independent repeats. **(D)** Transmigration of N931 (MOI 50) determined in UOK257 transfected with pLVTHM and UOK257 E-cadherin shRNA after 48 h of infection. Data represent the mean ± s.d with three independent repeats, ^*^P < 0.05.

We next tested if the delayed transmigration in UOK-FLCN cell was due to the defect in E-cadherin polarization. We, therefore, seeded control and E-cadherin knockdown cells on transwells. ZO-1 expression was not affected under these conditions (Figure 7A). The majority of the bacteria were on the apical side of the cells 24 h pi and more of them transmigrated with the progression of time until 96 h pi as seen on confocal microscopy of the transwell models infected with N931 at MOI 50 (Figure 7B). Infection did not affect the barrier integrity (Figure 7C). However, transmigration was strongly increased upon E-cadherin knockdown in the absence of FLCN expression (Figure 7D).

## Discussion

Although *N. gonorrhoeae* is mainly an extracellular pathogen, colonization of epithelial cell layers can lead to gonococcal invasion and transcytosis into the subepithelial space (Criss and Seifert, 2006; Wang et al., 2008). Gonococci in the subepithelial space have been detected in patients of disseminating gonococcal infection (Draper et al., 1980; McGee et al., 1981) suggesting that traversal of the epithelial layer is a relevant process in gonococcal pathogenesis. However, the mechanism of traversal is not understood in detail so far. Studies in polarized cell culture have shown that gonococci pass epithelial layers without breaching the tight junctions (Edwards et al., 2013). These findings suggest (but do not prove) that gonococci may use the intracellular route to overcome epithelial barriers. We investigated in this study the role of FLCN in the intracellular survival of gonococci and the traversal of these bacteria over epithelial barriers. Loss of FLCN on the one side affected the survival of intracellular gonococci by enhanced autophagy. This effect was not prominent in polarized epithelial cells in which FLCN was required to keep the epithelial barrier intact and prevent the traversal of gonococci.

Our initial observation of an impact of FLCN on the control of the autophagic flux and the intracellular survival of gonococci is in line with recent publications. Lu et al. (2019) demonstrated that gonococci are targeted to the autophagic pathway and are efficiently killed during the early phase after invasion. However, a subpopulation can survive autophagic killing and survives for extended periods. It would be interesting to test if FLCN is upregulated in these cells after long-term infections to downregulate the autophagic flux. In another study, the initiation of autophagy by gonococcal invaders was shown to depend on the interaction with the Type I membrane protein isoform CD46-cyt1 and its scaffold GOPC (Kim et al., 2019). CD46-cyt1/GOPC recruit VSP34/Beclin-1 and thereby induce autophagy (Meiffren et al., 2010). The downregulation of CD46-cyt1 and the remodeling of the lysosome later during infection promotes intracellular survival (Kim et al., 2019). Although it is not yet clear how lysosomal remodeling is orchestrated by gonococci, FLCN could be involved in this process. FLCN has been demonstrated to regulate lysosome function by promoting the mTORC1-dependent phosphorylation and cytoplasmic sequestration of the transcription factor EB (TFEB) (Petit et al., 2013), a master regulator for lysosome biogenesis (Sardiello et al., 2009) and autophagy (Settembre et al., 2011). FLCN thus prevents both lysosome formation and autophagy by interfering with these processes at the transcriptional level.

Based on the results obtained for a role of FLCN on intracellular survival of gonococci we assumed that traversal of epithelial layers should be prevented in the absence of FLCN if the intracellular route significantly contributes to this process. The opposite was the case. Gonococci traversed the epithelial layer more efficiently in the absence of FLCN. This effect was not simply due to the loss of epithelial integrity since the trans-epithelial permeability was very low even after extended times of infection, and irrespective of the absence or presence of FLCN. We, therefore, assumed that in these polarized epithelial cells another function of FLCN may be dominant, the control of cell polarity. FLCN has been shown to localize to adherence junctions by interacting with the beta-catenin homolog and armadillo repeat-containing protein p0071 (Medvetz et al., 2012; Nahorski et al., 2012). In line with a function in adherence junction formation, Goncharova et al. (2014) showed that loss of FLCN leads to downregulation of the major adherence junction protein E-cadherin in mouse alveolar epithelial cells. This is, however, in stark contrast to our findings since E-cadherin was strongly upregulated in UOK257 cells and downregulated in cells with restored FLCN expression (UOK-FLCN; see Figure 4). Whether the different findings of FLCN on E-cadherin expression depend on the cell type (airway/kidney) or the species (mouse/human) remains to be shown.

We found that E-cadherin is also involved in the control of gonococcal survival, however, the function appears to be different in polarized and non-polarized cells. In cells grown as 2D culture (non-polarized), a gonococcal infection caused an initial increase in E-cadherin expression which was downregulated at later time points. Interestingly, silencing E-cadherin expression in UOK257 cells significantly reduced the autophagic flux (Figure 5C) and strongly increased the survival of gonococci (Figure 5E). Since interfering with autophagy in E-cadherin expressing UOK257 cells rescued gonococcal survival, it is safe to say that E-cadherin functions as a positive regulator of autophagy in these non-polarized cells. In polarized cells, the function of E-cadherin is not as clear, and this has several reasons. Polarized cells are much more resilient to infection and assays have to be performed over days rather than for hours. It is therefore almost impossible to precisely determine the number of bacteria which enter these cells and survive in them. Gentamicin may not kill all extracellular bacteria, for example, bacteria may be protected if they are localized between cells below the tight junctions in an otherwise fully polarized epithelial layer. The clear effects we found for the transmigration of polarized epithelial cells are therefore hard to interpret. Clearly, loss of FLCN highly significantly increased transmigration over the epithelial layer whereas the gentamicin-resistant population was decreased, similarly as in non-polarized cells. This could indicate, that in polarized epithelial cells FLCN reduces extracellular traversal and increases intracellular survival.

Other than expected from the result obtained in non-polarized epithelial cells, E-cadherin silencing in high-expressing UOK257 cells boosted transmigration significantly (Figure 7D). A possible explanation for this observation may be that the loss of adherence junctions in the UOK257 cells upon E-cadherin knockdown facilitates gonococcal transmigration in otherwise intact polarized epithelial cells. This is in line with the results of a previous study in endometrial and fallopian tube epithelium which shows that gonococci induced redistribution of the adherens junction proteins E-cadherin and its adapter protein β-catenin from the membrane to a cytoplasmic pool. Similar as in our study (ZO-1), gonococcal infection did not induce modification or distribution of the tight junction proteins Occludin and ZO-1 (Rodríguez-Tirado et al., 2012). In primary polarized cells of the endocervix, infection with gonococci caused also the disruption of tight junctions and the redistribution of Occludin and ZO-1 (Edwards et al., 2013). It is, therefore, possible that disruption of junctional complexes is a more general mechanism of gonococcal traversal of polarized epithelia, which, depending on the tissue type may affect adherence and/or tight junctions.

FLCN has been connected to multiple cellular processes including mTOR signaling, transforming growth factor β (TGF- β), AMP-activated kinase (AMPK) signaling, autophagy and the formation of lysosomes. Intriguingly, all these pathways are also connected to cell polarization and integrity. Although FLCN has so far only been described in non-communicable diseases, it may play a more prominent role in infection than so far recognized. These functions may include the control of the epithelial barrier integrity and autophagy as defense strategies against invading pathogens.

## Data Availability Statement

All datasets generated for this study are included in the article/Supplementary Material.

## Author Contributions

TY, VK-P, and TR designed the experiments, TY conducted the experiments, TY, VK-P, and TR analyzed the results, MH provided material, MU and RH provided UOK257 and UOK-FLCN, and TY and TR wrote the manuscript. All authors contributed to the article and approved the submitted version.

## Conflict of Interest

The authors declare that the research was conducted in the absence of any commercial or financial relationships that could be construed as a potential conflict of interest.

## Supplementary Material

Figure S1 Schematic representation of plasmids pCAG-FLCN-SMAR. pCAG-FLCN-SMAR is used for generation of UOK-FLCN cell line. It contains the FLCN cDNA and harboring the S/MAR module downstream of the expression cassette. CAG, promoter; Puro, puromycin resistance gene; gray feature, P2A self-cleaving sequence; FLCN, FLCN cDNA; SMAR, scaffold/matrix attachment region; bb, bacterial backbone; Ele40, Element40 insulator.

Figure S2 FLCN didn't affect the Opa57 and PorB_IA_ mediated adherence nor survival. UOK cells infected with N313 (Opa57) in DMEM medium and N927 (PorB_IA_) in HEPEs respectively. Gentamicin protection assay was performed to determine the adherence and survival of bacteria. Data represent the mean ± s.d with three independent repeats.

Figure S3 FLCN downregulation leads to decreased survival of gonococci shown with absolute bacterial number. HeLa2000 and Hec-1-B cells and their respective knockdown cell clones were infected with N931 (MOI 50) in RPMI or DMEM medium with or without FBS and then subjected to a gentamicin protection assay. **(A)** Infected HeLa2000 cells were lysed the total number of colonies forming units was quantified by dilution plating. The left panel shows the number of adherent bacteria (calculated as total minus surviving bacteria). 50 μM gentamicin was added to the infected cells for 2 h to kill the extracellular gonococci. Cells were lysed and the number of colony forming units was quantified shown in the right panel. **(B)** Adherent (left panel) and intracellular (right panel) gonococci were quantified in Hela2000 cells with or without overexpression FLCN. **(C)** Adherent (left panel) and intracellular (right panel) bacteria were quantified in Hec-1-B control and FLCN knockdown cells in DMEM plus FBS. Data represent the mean ± s.d with three independent repeats. Significance was determined using student *t*-test, ^*^*P* < 0.05.

## References

[B1] Bos M. P. Grunert F. Belland R. J. (1997a). Differential recognition of members of the carcinoembryonic antigen family by Opa variants of *Neisseria gonorrhoeae*. Infect Immun 65:9. 10.1128/IAI.65.6.2353-2361.1997 9169774 PMC175326

[B2] Bos M. P. Hogan D. Belland R. J. (1997b). Selection of Opa^+^ *Neisseria gonorrhoeae* by limited availability of normal human serum. Infect Immun 65:6. 10.1128/IAI.65.2.645-650.1997 9009326 PMC176109

[B3] Chen T. (1995). Adherence of pilus^−^ Opa^+^ gonococci to epithelial cells *in vitro* involves heparan sulfate. J. Exp. Med. 182, 511–517. 10.1084/jem.182.2.511 7629509 PMC2192128

[B4] Chen T. Gotschlich E. C. (1996). CGM1a antigen of neutrophils, a receptor of gonococcal opacity proteins. Proc. Natl. Acad. Sci. U.S.A. 93, 14851–14856. 10.1073/pnas.93.25.14851 8962144 PMC26225

[B5] Criss A. K. Seifert H. S. (2006). Gonococci exit apically and basally from polarized epithelial cells and exhibit dynamic changes in type IV pili. Cell. Microbiol. 8, 1430–1443. 10.1111/j.1462-5822.2006.00722.x 16922862 PMC2290004

[B6] Dehio M. Gómez-Duarte O. G. Dehio C. Meyer T. F. (1998). Vitronectin-dependent invasion of epithelial cells by *Neisseria gonorrhoeae* involves α integrin receptors. FEBS Lett. 424, 84–88. 10.1016/S0014-5793(98)00144-6 9537520

[B7] Draper D. L. James J. F. Brooks G. F. Sweet R. L. (1980). Comparison of virulence markers of peritoneal and fallopian tube isolates with endocervical *Neisseria gonorrhoeae* isolates from women with acute salpingitis. Infect. Immun. 27, 882–888. 10.1128/IAI.27.3.882-888.1980 6769811 PMC550857

[B8] Duensing T. D. van Putten J. P. M. (1998). Vitronectin binds to the gonococcal adhesin OpaA through a glycosaminoglycan molecular bridge. Biochem. J. 334, 133–139. 10.1042/bj3340133 9693112 PMC1219671

[B9] Dunlop E. A. Seifan S. Claessens T. Behrends C. Kamps M. A. Rozycka E.. (2014). FLCN, a novel autophagy component, interacts with GABARAP and is regulated by ULK1 phosphorylation. Autophagy 10, 1749–1760. 10.4161/auto.29640 25126726 PMC4198360

[B10] Edwards V. L. Wang L.-C. Dawson V. Stein D. C. Song W. (2013). *Neisseria gonorrhoeae* breaches the apical junction of polarized epithelial cells for transmigration by activating EGFR: Gonococci breach the apical junction of epithelia. Cell. Microbiol. 15, 1042–1057. 10.1111/cmi.12099 23279089 PMC5584544

[B11] Faulstich M. Böttcher J.-P. Meyer T. F. Fraunholz M. Rudel T. (2013). Pilus phase variation switches gonococcal adherence to invasion by caveolin-1-dependent host cell signaling. PLoS Pathog. 9:e1003373. 10.1371/journal.ppat.1003373 23717204 PMC3662692

[B12] Goncharova E. A. Goncharov D. A. James M. L. Atochina-Vasserman E. N. Stepanova V. Hong S.-B.. (2014). Folliculin controls lung alveolar enlargement and epithelial cell survival through E-Cadherin, LKB1, and AMPK. Cell Rep. 7, 412–423. 10.1016/j.celrep.2014.03.025 24726356 PMC4034569

[B13] Gray-Owen S. D. (1997). CD66 carcinoembryonic antigens mediate interactions between Opa-expressing *Neisseria gonorrhoeae* and human polymorphonuclear phagocytes. EMBO J. 16, 3435–3445. 10.1093/emboj/16.12.3435 9218786 PMC1169969

[B14] Gray-Owen S. D. Lorenzen D. R. Haude A. Meyer T. F. Dehio C. (1997). Differential Opa specificities for CD66 receptors influence tissue interactions and cellular response to *Neisseria gonorrhoeae*. Mol. Microbiol. 26, 971–980. 10.1046/j.1365-2958.1997.6342006.x 9426134

[B15] Hartsock A. Nelson W. J. (2008). Adherens and tight junctions: Structure, function and connections to the actin cytoskeleton. Biochim. Biophys. Acta (BBA) Biomembr. 1778, 660–669. 10.1016/j.bbamem.2007.07.012 17854762 PMC2682436

[B16] Heydarian M. Yang T. Schweinlin M. Steinke M. Walles H. Rudel T.. (2019). Biomimetic human tissue model for long-term study of *Neisseria gonorrhoeae* infection. Front. Microbiol. 10:e1740. 10.3389/fmicb.2019.01740 31417529 PMC6685398

[B17] Kepp O. Rajalingam K. Kimmig S. Rudel T. (2007). Bak and Bax are non-redundant during infection- and DNA damage-induced apoptosis. EMBO J. 26, 825–834. 10.1038/sj.emboj.7601533 17235284 PMC1794390

[B18] Kim W. J. Mai A. Weyand N. J. Rendón M. A. Van Doorslaer K. So M. (2019). *Neisseria gonorrhoeae* evades autophagic killing by downregulating CD46-cyt1 and remodeling lysosomes. PLOS Pathog. 15:e1007495. 10.1371/journal.ppat.1007495 30753248 PMC6388937

[B19] Kuhlewein C. Rechner C. Meyer T. F. Rudel T. (2006). Low-phosphate-dependent invasion resembles a general way for *Neisseria gonorrhoeae* to enter host cells. Infect. Immun. 74, 4266–4273. 10.1128/IAI.00215-06 16790801 PMC1489691

[B20] Kupsch E. M. Knepper B. Kuroki T. Heuer I. Meyer T. F. (1993). Variable opacity (Opa) outer membrane proteins account for the cell tropisms displayed by *Neisseria gonorrhoeae* for human leukocytes and epithelial cells. EMBO J. 12, 641–650. 10.1002/j.1460-2075.1993.tb05697.x 8440254 PMC413248

[B21] Łaniewski P. Gomez A. Hire G. So M. Herbst-Kralovetz M. M. (2017). Human three-dimensional endometrial epithelial cell model to study host interactions with vaginal bacteria and *Neisseria gonorrhoeae*. Infect. Immun. 85:16. 10.1128/IAI.01049-16 28052997 PMC5328489

[B22] Laviolette L. A. Mermoud J. Calvo I. A. Olson N. Boukhali M. Steinlein O. K.. (2017). Negative regulation of EGFR signalling by the human folliculin tumour suppressor protein. Nat. Commun. 8:15866. 10.1038/ncomms15866 28656962 PMC5493755

[B23] Lin L. Ayala P. Larson J. Mulks M. Fukuda M. Carlsson S. R.. (1997). The *Neisseria* type 2 IgA1 protease cleaves LAMP1 and promotes survival of bacteria within epithelial cells. Mol. Microbiol. 24, 1083–1094. 10.1046/j.1365-2958.1997.4191776.x 9220014

[B24] Lu P. Wang S. Lu Y. Neculai D. Sun Q. van der Veen S. (2019). A subpopulation of intracellular *Neisseria gonorrhoeae* escapes autophagy-mediated killing inside epithelial cells. J. Infect. Dis. 219, 133–144. 10.1093/infdis/jiy237 29688440

[B25] Makino S. van Putten J. P. Meyer T. F. (1991). Phase variation of the opacity outer membrane protein controls invasion by *Neisseria gonorrhoeae* into human epithelial cells. EMBO J. 10, 1307–1315. 10.1002/j.1460-2075.1991.tb07649.x 1673923 PMC452788

[B26] McGee Z. A. Johnson A. P. Taylor-Robinson D. (1981). Pathogenic mechanisms of *Neisseria gonorrhoeae*: observations on damage to human fallopian tubes in organ culture by gonococci of colony type 1 or type 4. J. Infect. Dis. 143, 413–422. 10.1093/infdis/143.3.413 6785363

[B27] Medvetz D. A. Khabibullin D. Hariharan V. Ongusaha P. P. Goncharova E. A. Schlechter T.. (2012). Folliculin, the product of the Birt-Hogg-Dube tumor suppressor gene, interacts with the adherens junction protein p0071 to regulate cell-cell adhesion. PLoS ONE 7:e47842. 10.1371/journal.pone.0047842 23139756 PMC3490959

[B28] Meiffren G. Joubert P.-E. Grégoire I. P. Codogno P. Rabourdin-Combe C. Faure M. (2010). Pathogen recognition by the cell surface receptor CD46 induces autophagy. Autophagy 6, 299–300. 10.4161/auto.6.2.11132 20087059

[B29] Miyoshi J. Takai Y. (2005). Molecular perspective on tight-junction assembly and epithelial polarity. Adv. Drug Deliv. Rev. 57, 815–855. 10.1016/j.addr.2005.01.008 15820555

[B30] Morello J. A. Bohnhoff M. (1989). Serovars and serum resistance of *Neisseria gonorrhoeae* from disseminated and uncomplicated infections. J. Infect. Dis. 160, 1012–1017. 10.1093/infdis/160.6.1012 2511251

[B31] Muller A. (2002). VDAC and the bacterial porin PorB of *Neisseria gonorrhoeae* share mitochondrial import pathways. EMBO J. 21, 1916–1929. 10.1093/emboj/21.8.1916 11953311 PMC125974

[B32] Nahorski M. S. Seabra L. Straatman-Iwanowska A. Wingenfeld A. Reiman A. Lu X. (2012). Folliculin interacts with p0071 (plakophilin-4) and deficiency is associated with disordered RhoA signalling, epithelial polarization and cytokinesis. Hum. Mol. Genet. 21, 5268–5279. 10.1093/hmg/dds378 22965878 PMC3755511

[B33] Niessen C. M. Gottardi C. J. (2008). Molecular components of the adherens junction. Biochim. Biophys. Acta BBA Biomembr. 1778, 562–571. 10.1016/j.bbamem.2007.12.015 18206110 PMC2276178

[B34] Nighot P. Ma T. (2016). Role of autophagy in the regulation of epithelial cell junctions. Tissue Barriers 4:e1171284. 10.1080/21688370.2016.1171284 27583189 PMC4993570

[B35] Petit C. S. Roczniak-Ferguson A. Ferguson S. M. (2013). Recruitment of folliculin to lysosomes supports the amino acid–dependent activation of Rag GTPases. J. Cell Biol. 202, 1107–1122. 10.1083/jcb.201307084 24081491 PMC3787382

[B36] Possik E. Jalali Z. Nouët Y. Yan M. Gingras M.-C. Schmeisser K.. (2014). Folliculin regulates Ampk-Dependent autophagy and metabolic stress survival. PLoS Genet. 10:e1004273. 10.1371/journal.pgen.1004273 24763318 PMC3998892

[B37] Rechner C. Kühlewein C. Müller A. Schild H. Rudel T. (2007). Host glycoprotein Gp96 and scavenger receptor SREC interact with PorB of disseminating *Neisseria gonorrhoeae* in an epithelial invasion pathway. Cell Host Microbe 2, 393–403. 10.1016/j.chom.2007.11.002 18078691

[B38] Rodríguez-Tirado C. Maisey K. Rodríguez F. E. Reyes-Cerpa S. Reyes-López F. E. Imarai M. (2012). *Neisseria gonorrhoeae* induced disruption of cell junction complexes in epithelial cells of the human genital tract. Microbes Infection 14, 290–300. 10.1016/j.micinf.2011.11.002 22146107

[B39] Rudel T. Putten J. P. M. Gibbs C. P. Haas R. Meyer T. F. (1992). Interaction of two variable proteins (PilE and PilC) required for pilus-mediated adherence of *Neisseria gonorrhoeae* to human epithelial cells. Mol. Microbiol. 6, 3439–3450. 10.1111/j.1365-2958.1992.tb02211.x 1362447

[B40] Sardiello M. Palmieri M. di Ronza A. Medina D. L. Valenza M. Gennarino V. A.. (2009). A gene network regulating lysosomal biogenesis and function. Science 325, 473–477. 10.1126/science.1174447 19556463

[B41] Schmidt L. S. Linehan W. M. (2018). FLCN : the causative gene for Birt-Hogg-Dubé syndrome. Gene 640, 28–42. 10.1016/j.gene.2017.09.044 28970150 PMC5682220

[B42] Settembre C. Di Malta C. Polito V. A. Arencibia M. G. Vetrini F. Erdin S.. (2011). TFEB links autophagy to lysosomal biogenesis. Science 332, 1429–1433. 10.1126/science.1204592 21617040 PMC3638014

[B43] Skolnik K. Tsai W. H. Dornan K. Perrier R. Burrowes P. W. Davidson W. J. (2016). Birt-Hogg-Dubé syndrome: a large single family cohort. Respir. Res. 17:22. 10.1186/s12931-016-0339-2 26928018 PMC4770529

[B44] Starling G. P. Yip Y. Y. Sanger A. Morton P. E. Eden E. R. Dodding M. P. (2016). Folliculin directs the formation of a Rab34–RILP complex to control the nutrient-dependent dynamic distribution of lysosomes. EMBO Rep. 17, 823–841. 10.15252/embr.201541382 27113757 PMC4893818

[B45] Stein D. C. LeVan A. Hardy B. Wang L.-C. Zimmerman L. Song W. (2015). Expression of Opacity proteins interferes with the transmigration of *Neisseria gonorrhoeae* across polarized epithelial Cells. PLoS ONE 10:e0134342. 10.1371/journal.pone.0134342 26244560 PMC4526573

[B46] van Putten J. P. Paul S. M. (1995). Binding of syndecan-like cell surface proteoglycan receptors is required for *Neisseria gonorrhoeae* entry into human mucosal cells. EMBO J. 14, 2144–2154. 10.1002/j.1460-2075.1995.tb07208.x 7774572 PMC398320

[B47] van Putten J. P. M. (1998). Gonococcal invasion of epithelial cells driven by P.IA, a bacterial ion channel with GTP binding properties. J. Exp. Med. 188, 941–952. 10.1084/jem.188.5.941 9730895 PMC2213401

[B48] Virji M. Makepeace K. Ferguson D. J. P. Watt S. M. (1996). Carcinoembryonic antigens (CD66) on epithelial cells and neutrophils are receptors for Opa proteins of pathogenic neisseriae. Mol. Microbiol. 22, 941–950. 10.1046/j.1365-2958.1996.01551.x 8971715

[B49] Wang J. A. Meyer T. F. Rudel T. (2008). Cytoskeleton and motor proteins are required for the transcytosis of *Neisseria gonorrhoeae* through polarized epithelial cells. Int. J. Med. Microbiol. 298, 209–221. 10.1016/j.ijmm.2007.05.004 17683982

[B50] Wang L.-C. Yu Q. Edwards V. Lin B. Qiu J. Turner J. R.. (2017). *Neisseria gonorrhoeae* infects the human endocervix by activating non-muscle myosin II-mediated epithelial exfoliation. PLoS Pathog 13:e1006269. 10.1371/journal.ppat.1006269 28406994 PMC5391109

[B51] Wira C. R. Fahey J. V. Sentman C. L. Pioli P. A. Shen L. (2005). Innate and adaptive immunity in female genital tract: cellular responses and interactions. Immunol. Rev. 206, 306–335. 10.1111/j.0105-2896.2005.00287.x 16048557

[B52] Wong S.-P. Harbottle R. P. (2013). Genetic modification of dividing cells using episomally maintained S/MAR DNA vectors. Mol. Ther. Nucleic Acids 2:e115. 10.1038/mtna.2013.40 23941867 PMC3759738

[B53] Yang Y. Padilla-Nash H. M. Vira M. A. Abu-Asab M. S. Val D. Worrell R.. (2008). The UOK 257 cell line: a novel model for studies of the human Birt–Hogg–Dubé gene pathway. Cancer Genetics Cytogenetics 180, 100–109. 10.1016/j.cancergencyto.2007.10.010 18206534 PMC2440670

[B54] Yoshii S. R. Mizushima N. (2017). Monitoring and measuring autophagy. IJMS 18, 1865. 10.3390/ijms18091865 28846632 PMC5618514

[B55] Zhao L. Ji X. Zhang X. Li L. Jin Y. Liu W. (2018). FLCN is a novel Rab11A-interacting protein that is involved in the Rab11A-mediated recycling transport. J. Cell Sci. 131:jcs218792. 10.1242/jcs.218792 30446510

