# Supplementary figures and images for: Folliculin Controls the Intracellular Survival and Trans-Epithelial Passage of Neisseria gonorrhoeae
Source: Front Cell Infect Microbiol. 2020 Sep 4;10:422. doi: 10.3389/fcimb.2020.00422 (PMC7499807; doi:10.3389/fcimb.2020.00422)

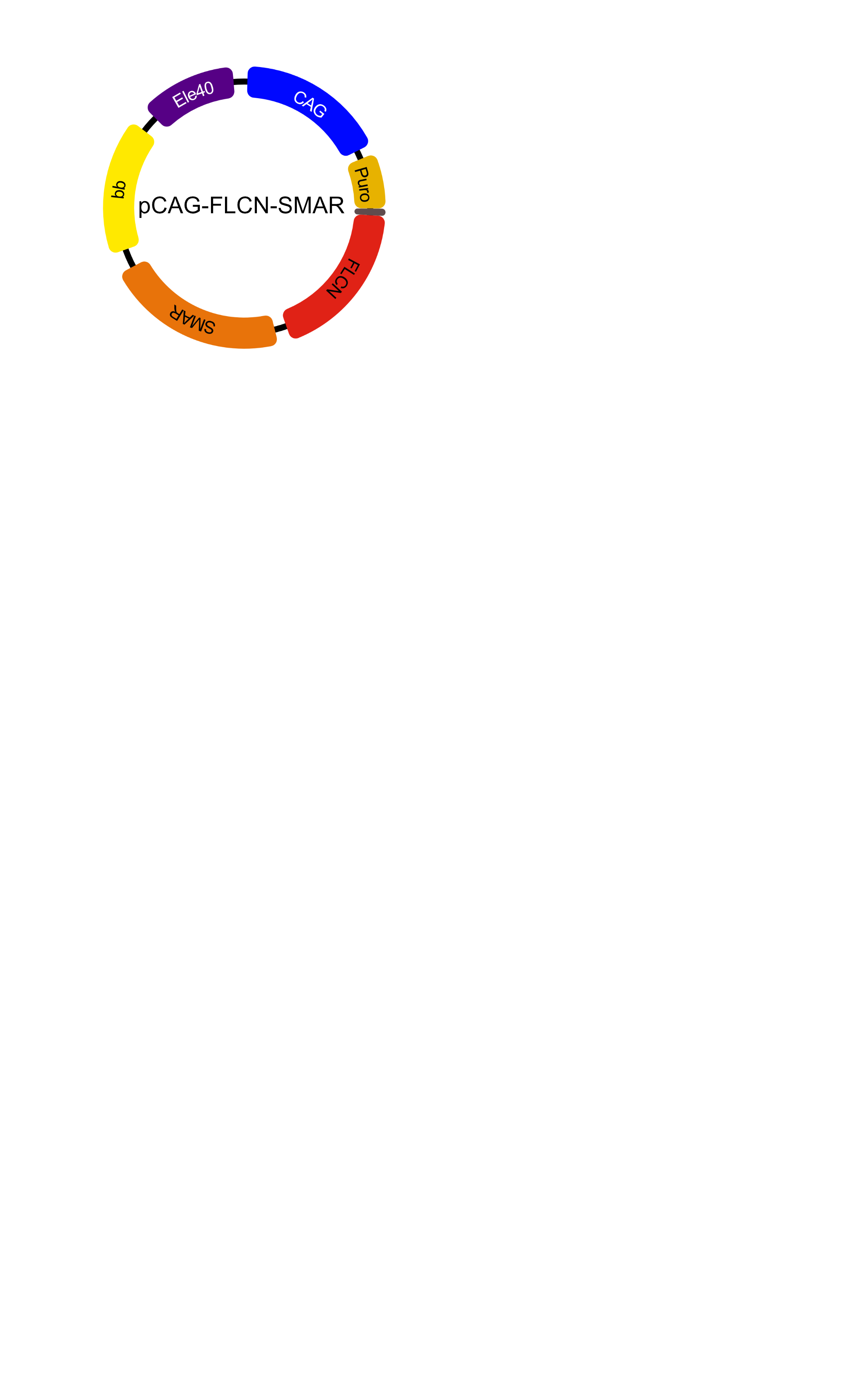

Supplement: Figure S1 — Schematic representation of plasmids pCAG-FLCN-SMAR. pCAG-FLCN-SMAR is used for generation of UOK-FLCN cell line. It contains the FLCN cDNA and harboring the S/MAR module downstream of the expression cassette. CAG, promoter; Puro, puromycin resistance gene; gray feature, P2A self-cleaving sequence; FLCN, FLCN cDNA; SMAR, scaffold/matrix attachment region; bb, bacterial backbone; Ele40, Element40 insulator. [file Image_1.JPEG]

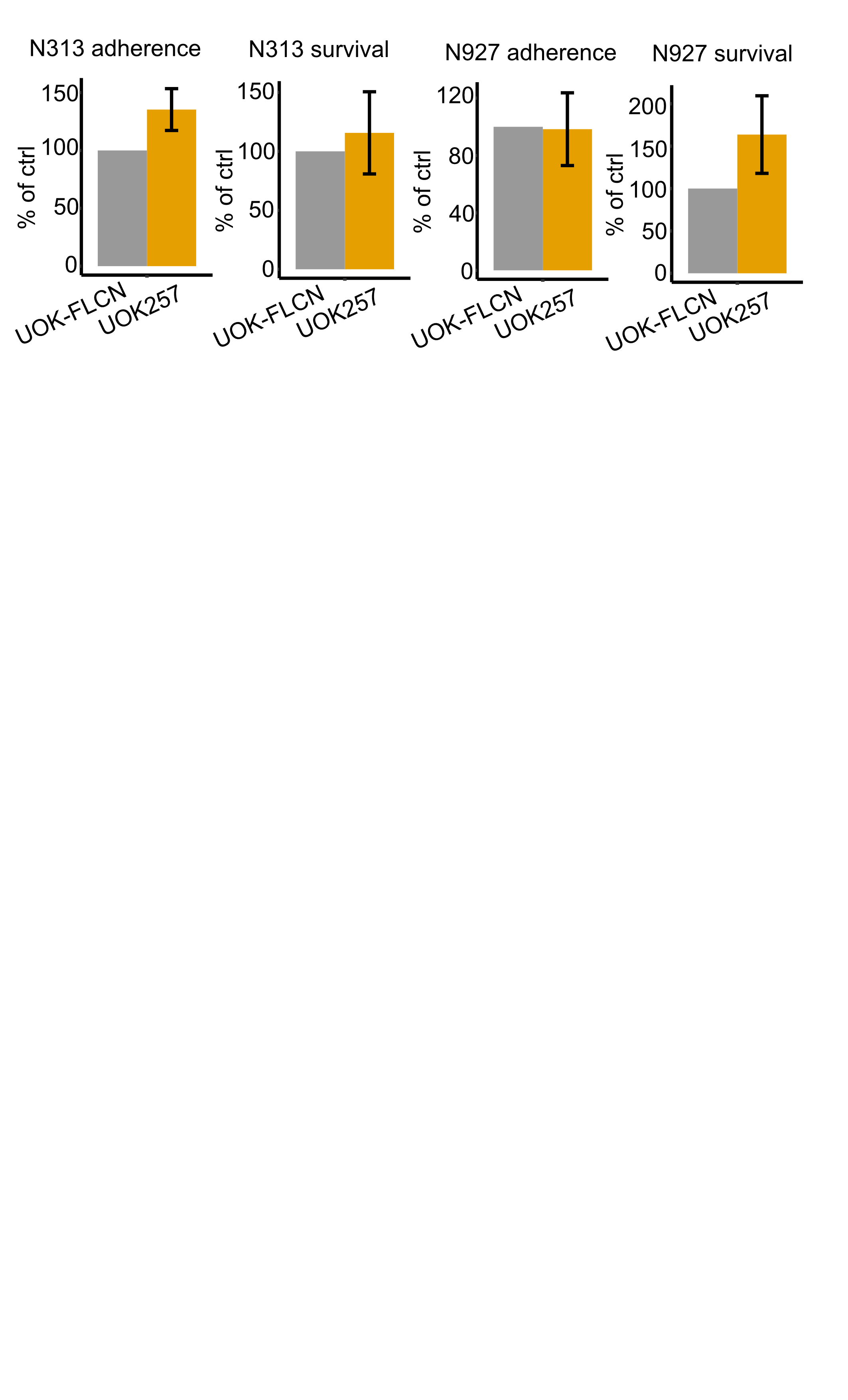

Supplement: Figure S2 — FLCN didn't affect the Opa57 and PorBIA mediated adherence nor survival. UOK cells infected with N313 (Opa57) in DMEM medium and N927 (PorBIA) in HEPEs respectively. Gentamicin protection assay was performed to determine the adherence and survival of bacteria. Data represent the mean ± s.d with three independent repeats. [file Image_2.JPEG]

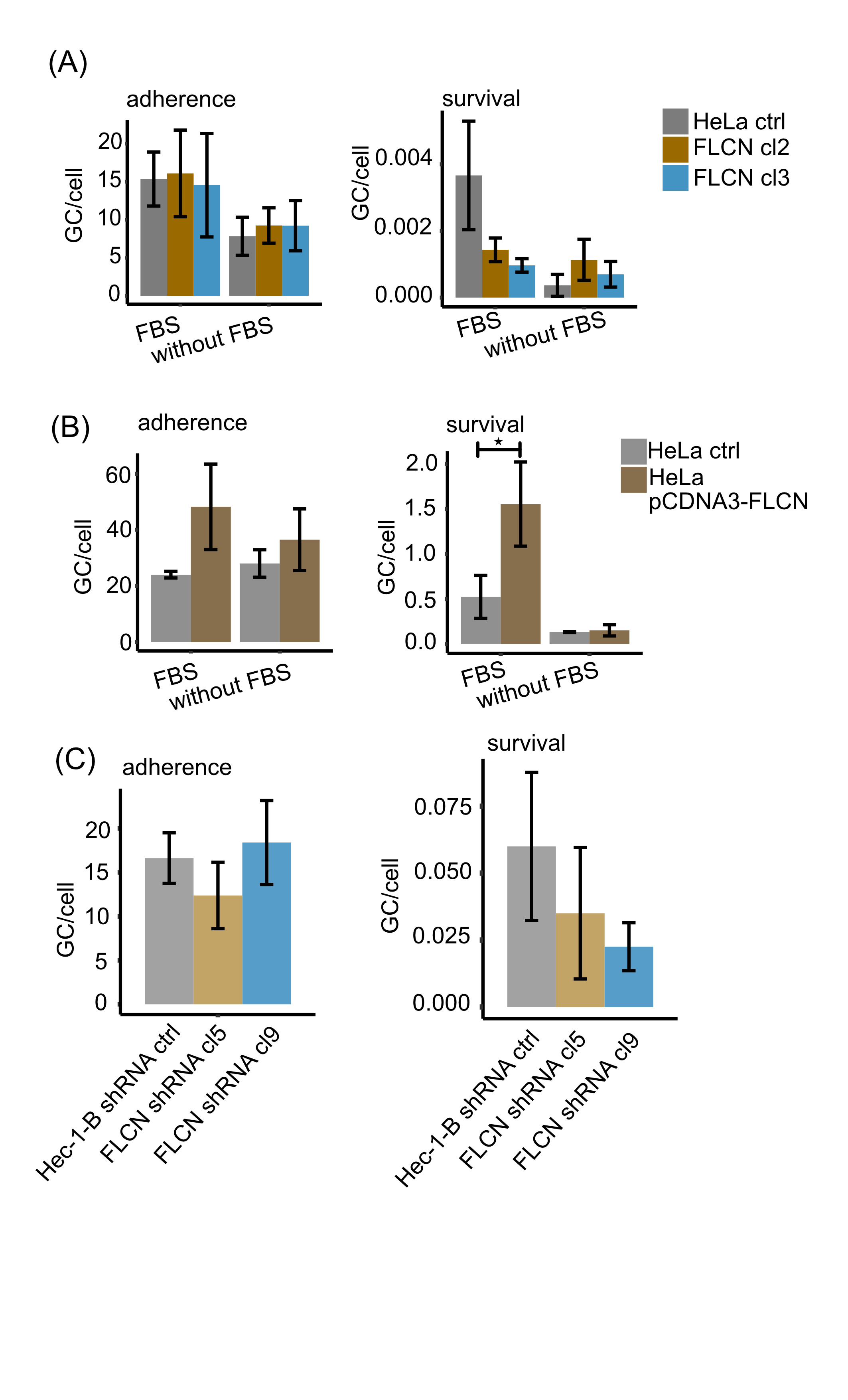

Supplement: Figure S3 — FLCN downregulation leads to decreased survival of gonococci shown with absolute bacterial number. HeLa2000 and Hec-1-B cells and their respective knockdown cell clones were infected with N931 (MOI 50) in RPMI or DMEM medium with or without FBS and then subjected to a gentamicin protection assay. (A) Infected HeLa2000 cells were lysed the total number of colonies forming units was quantified by dilution plating. The left panel shows the number of adherent bacteria (calculated as total minus surviving bacteria). 50 μM gentamicin was added to the infected cells for 2 h to kill the extracellular gonococci. Cells were lysed and the number of colony forming units was quantified shown in the right panel. (B) Adherent (left panel) and intracellular (right panel) gonococci were quantified in Hela2000 cells with or without overexpression FLCN. (C) Adherent (left panel) and intracellular (right panel) bacteria were quantified in Hec-1-B control and FLCN knockdown cells in DMEM plus FBS. Data represent the mean ± s.d with three independent repeats. Significance was determined using student t-test, *P < 0.05. [file Image_3.JPEG]
